# A metabolite-centric view on flux distributions in genome-scale metabolic models

**DOI:** 10.1186/1752-0509-7-33

**Published:** 2013-04-12

**Authors:** S Alexander Riemer, René Rex, Dietmar Schomburg

**Affiliations:** 1 Department of Bioinformatics and Biochemistry, Technische Universität Braunschweig, Braunschweig, Germany

**Keywords:** Split ratios, Branch points, Metabolic modelling, Flux balance analysis, Metabolic reconstruction, Constraint-based modelling, Stoichiometric matrix, Linear programming, iJO1366, iAF1260

## Abstract

**Background:**

Genome-scale metabolic models are important tools in systems biology. They permit the *in-silico* prediction of cellular phenotypes via mathematical optimisation procedures, most importantly flux balance analysis. Current studies on metabolic models mostly consider reaction fluxes in isolation. Based on a recently proposed metabolite-centric approach, we here describe a set of methods that enable the analysis and interpretation of flux distributions in an integrated metabolite-centric view. We demonstrate how this framework can be used for the refinement of genome-scale metabolic models.

**Results:**

We applied the metabolite-centric view developed here to the most recent metabolic reconstruction of *Escherichia coli*. By compiling the balance sheets of a small number of currency metabolites, we were able to fully characterise the energy metabolism as predicted by the model and to identify a possibility for model refinement in NADPH metabolism. Selected branch points were examined in detail in order to demonstrate how a metabolite-centric view allows identifying functional roles of metabolites. Fructose 6-phosphate aldolase and the sedoheptulose bisphosphate bypass were identified as enzymatic reactions that can carry high fluxes in the model but are unlikely to exhibit significant activity *in vivo*. Performing a metabolite essentiality analysis, unconstrained import and export of iron ions could be identified as potentially problematic for the quality of model predictions.

**Conclusions:**

The system-wide analysis of split ratios and branch points allows a much deeper insight into the metabolic network than reaction-centric analyses. Extending an earlier metabolite-centric approach, the methods introduced here establish an integrated metabolite-centric framework for the interpretation of flux distributions in genome-scale metabolic networks that can complement the classical reaction-centric framework. Analysing fluxes and their metabolic context simultaneously opens the door to systems biological interpretations that are not apparent from isolated reaction fluxes. Particularly powerful demonstrations of this are the analyses of the complete metabolic contexts of energy metabolism and the folate-dependent one-carbon pool presented in this work. Finally, a metabolite-centric view on flux distributions can guide the refinement of metabolic reconstructions for specific growth scenarios.

## Background

The advent of rapid sequencing technologies, the collection and sharing of omics data in electronic databases, and the development of a wealth of computational tools in systems biology have allowed the reconstruction of metabolic networks on a genome-wide scale. Since the first whole-genome metabolic model was published in 1999 [1], the number of genome-scale metabolic reconstructions has been growing steadily, and a recent review lists metabolic reconstructions for more than sixty organisms [2].

Whole-genome metabolic models play a dual role in systems biology: Firstly, they are structured knowledge bases that integrate data about the metabolism of the modelled organism from a wide variety of sources, including databases, primary literature, and expert knowledge [3]. Secondly, they can be used to predict cellular phenotypes, which allows, inter alia, the *in-silico* prediction of the effects of genetic or regulatory modifications or growth in different environments on the phenotype [4]. Thus, they are useful tools for generating hypotheses to guide wet-lab experiments and for providing context for the interpretation of experimental data. In addition, metabolic models are needed for metabolic engineering, the directed modification of the organism with respect to a metabolic goal such as overproduction and export of some industrially relevant chemical compound [5].

*Escherichia coli* is by far the best-studied prokaryote and an important model organism in biology. Its metabolism is particularly well understood, and many enzymes and biochemical pathways were first described in *E. coli* (e.g. [6-9]). The latest whole-genome metabolic reconstruction of *E. coli* K-12 MG1655, designated iJO1366 [10], is one of the most comprehensive genome-scale metabolic models available. It is an extension of the earlier models iAF1260 [11] and iJR904 [12].

Flux balance analysis and related methods for the analysis of whole-genome metabolic models produce a vector of reaction fluxes. These are usually either given as absolute values in mmol gDW^−1^ h^−1^ or as percentage of the substrate uptake rate. Such values are, however, very hard to interpret. For instance, what does it mean if the flux from 2-oxoglutarate to L-glutamate via glutamate dehydrogenase is 6.3 mmol gDW^−1^ h^−1^? Or that the rate of aspartate formation via aspartate transaminase is 22% of the glucose uptake rate?

All genome-scale metabolic models published to date are stoichiometric models, which incorporate reaction stoichiometry but do not require any knowledge of kinetic parameters. While they do not allow the analysis of non-equilibrium dynamics, stoichiometric models have been found very useful for predicting the metabolic capabilities in steady state [12-15].

Flux balance analysis (FBA) was introduced in 1986 [16], more than a decade before the first genome-scale metabolic reconstruction, and has become the most commonly used method for predicting steady-state fluxes in metabolic networks. Its core idea is the assumption that wild-type organisms have been optimised through the course of their evolution [17], e.g. for rapid or efficient growth.

Oberhardt et al. [4] list the following major goals commonly pursued in analyses of whole-genome metabolic reconstructions: (1) Contextualisation of high-throughput data, (2) guidance of metabolic engineering, (3) directing hypothesis-driven discovery, (4) interrogation of multi-species relationships, and (5) network property discovery. Analyses pertaining to all of these goals are currently performed chiefly in a reaction-centric framework.

Contextualisation of high-throughput data is achieved by adding constraints to the model or modifying existing constraints [4]. Usually, these changes only pertain to isolated reaction fluxes. For instance, both a gene knockout and the regulatory suppression of a gene product are modelled by constraining the flux through all reactions catalysed by the gene product to zero [18]. Of particular importance are gene expression data, which are also usually incorporated in the form of constraints on individual reaction fluxes [19-22]. Furthermore, rapid phenotyping data (such as from BIOLOG phenotype microarrays) are employed in the form of binary variables (growth/ no growth on a particular substrate) [15,23].

While the performance of models may initially be analysed in terms of growth rate and efficiency, the most specific tool for the validation of metabolic models is the comparison of predicted flux distributions to experimentally determined fluxes, usually obtained via ^13^C-labeling experiments [11,24]. Due to the lower concentrations of metabolites in peripheral pathways, this method is limited to fluxes in or close to central carbon metabolism [25-27].

Model-based metabolic engineering also employs a reaction-centric view. The most commonly used approaches try to modify individual reaction fluxes to redirect metabolic flux or to find a combination of gene knockouts leading to overproduction of the desired metabolite [28-31].

A first metabolite-centric approach for the analysis of genome-scale metabolic models was introduced in 2007 [32] and expanded in 2009 [33]. This approach, termed flux-sum analysis, has been employed successfully for the prediction of metabolite essentiality and for studying the robustness of metabolic networks to perturbations in metabolite turnover rates. To our knowledge, its application has so far been limited to the study of network robustness, either as a fundamental network property [33] or with the specific goal of identifying novel drug targets [34].

The concept of branch points and flux split ratios has been used in wet-lab metabolic flux analysis using small-scale metabolic models [35-37], particularly in the context of metabolic engineering projects [38-40]. There also exist implementations of FBA that explicitly support linear constraints defined in terms of split ratios [41,42]. However, to our knowledge, this concept has never been applied systematically for the analysis of the (predicted) flux distribution in a whole-genome metabolic reconstruction.

Here we show that the system-wide analysis of split ratios and branch points, leading to a metabolite-centric view on flux distributions, allows a much deeper insight into the metabolic network based on the results of flux balance analysis. For this purpose, we introduce a set of methods that generate a metabolite-centric description based on a given flux distribution. Moreover, we demonstrate how a system-wide analysis of metabolite essentiality, which is only possible within a metabolite-centric framework, can identify potential problems in a network reconstruction. We further show how, e.g., energy metabolism can be characterised by examining the flux split ratios for a very small number of metabolite nodes. We also demonstrate how a metabolite-centric view can be used for the refinement of models against experimental data, in particular by identifying enzymatic reactions not known to occur *in vivo* or known for very specific scenarios only, i.e. reactions or enzymes whose presence in the model may lower the quality of the model’s predictions in some, possibly most, scenarios.

## Results

FBA was performed for the chosen scenario, carbon-limited aerobic growth in glucose minimal medium with forced acetate secretion (see Methods section), with the following constraints: Glucose uptake was limited to a rate of at most 11.0 mmol gDW^−1^ h^−1^, and a lower bound of 6.4 mmol gDW^−1^ h^−1^ was imposed on the acetate export flux. For this scenario, FBA predicted a growth rate of 0.85 h^−1^, an oxygen uptake rate of 16.5 mmol gDW^−1^ h^−1^, and a CO_2_ emission rate of 18.2 mmol gDW^−1^ h^−1^. In the FBA solution, only 552 of the 2583 reactions (21%) involving 557 of the 1805 metabolites (31%) are active (at a cutoff of 10^−12^ mmol gDW^−1^ h^−1^). Of the 557 active metabolites, 401 are part of unbranched paths, i.e. they are each produced via a single reaction and consumed via another single reaction. These metabolites are purely transitory in the scenario under consideration. Consequently, only the remaining 156 metabolites (8.6% of all metabolite nodes or 28% of the active metabolites) are branch points, where fluxes split or join.

### Balance sheets of currency metabolites

Split-ratio analysis was used to explore the fate of currency metabolites, most importantly energy-rich phosphates (such as ATP, GTP, and phosphoenolpyruvate (PEP)) and reducing equivalents (chiefly NADH and NADPH).

#### ATP

Table 1 lists the relative contributions of reactions producing ATP. The total flux through ATP is 69.7 mmol gDW^−1^ h^−1^, which has to be corrected for salvage reactions, such as for diphosphate, which regenerate some “spent” ATP. This correction yields an ATP production rate of 66.9 mmol gDW^−1^ h^−1^, i.e. the net yield is 6.1 molecules of ATP per glucose molecule. The largest part (61.4% of the total ATP flux) is produced via oxidative phosphorylation. 25.5% is produced in glycolysis, while 8.5% is produced in the conversion of acetyl-CoA to acetate, which is excreted. While 4.0% of the total producing ATP flux stems from the reaction of ADP with diphosphate, only 0.7% is produced in the TCA cycle.

**Table 1:** Split ratios of ATP-producing fluxes

**Producing fluxes**		
**Reaction/Enzyme**	**Pathway/Product**	**Ratio**
ATP synthase	oxidative phosphorylation	61.35%
phosphoglycerate kinase	glycolysis	25.47%
acetate kinase	acetate export	8.46%
polyphosphate kinase	ADP + diphosphate	4.02%
succinyl-CoA synthetase	TCA cycle	0.71%

Total flux: 69.7 mmol gDW^−1^ h^−1^. Ratios below 0.05% not shown.

The ratios of ATP-consuming fluxes are shown in Table 2. Most of the ATP is consumed in the biomass reaction as growth-associated maintenance requirement (GAM; see Methods) (66.2%), fuelling growth-related processes. The second largest fraction is glycolysis, which consumes 8.8% and thus has an overall positive ATP balance. 6.2% is used in the biosynthesis of L-amino acids, one-third of which is accounted for by L-glutamine alone (2.1% of the ATP flux), followed by L-threonine (1.3%) and L-arginine (1.1%). The non-growth associated maintenance requirement (NGAM; see Methods) accounts for 4.5% of the total ATP consumption, purine metabolism consumes 3.4%, and 2.7% is used for the (re)phosphorylation of AMP to ADP. Another 2.7% is consumed in fatty acid biosynthesis, while 1.6% is converted to the other nucleoside triphosphates GTP (0.9%), UTP (0.5%), and CTP (0.2%). 1.2% of the ATP is spent in pyrimidine metabolism, and 1.1% is used in the pentose phosphate pathway for the production of phosphoribosyl pyrophosphate, while another 0.6% is consumed in sulfate assimilation. 0.2% of the total ATP enters the biomass directly. The rest is used in the biosynthesis of other biomass components, including cell wall components (peptidoglycans), deoxyribonucleoside triphosphates, glycogen, lipopolysaccharides, phospholipids, and various cofactors.

**Table 2:** Split ratios of ATP-consuming fluxes

**Consuming fluxes**		
**Reaction/Enzyme**	**Pathway/Product**	**Ratio**
Biomass (incl. GAM)		66.39%
phosphofructokinase	glycolysis	8.77%
ATP maintenance requirement	NGAM	4.52%
adenylate kinase	AMP→ADP	2.73%
acetyl-CoA carboxylase	fatty acids	2.68%
AICAR biosynthesis from PRPP (5 reactions)	purines	2.67%
glutamine synthetase	Gln	2.13%
aspartate kinase	Thr, Met, Lys, peptidoglycans	1.28%
PRPP synthetase	pentose phosphate pathway	1.11%
nucleoside-diphosphate kinase	GTP	0.92%
carbamate kinase	pyrimidines, Arg, polyamines	0.75%
homoserine kinase	Thr	0.65%
nucleoside-diphosphate kinase	UTP	0.49%
shikimate kinase	shikimate pathway	0.46%
UMP kinase	pyrimidines	0.44%
acetylglutamate kinase	Arg, polyamines	0.40%
argininosuccinate synthase	Arg	0.35%
adenylyl-sulfate kinase	sulfur metabolism	0.30%
sulfate adenyltransferase	sulfur metabolism	0.30%
guanylate kinase	purines	0.29%
GMP synthase	purines	0.29%
asparagine synthetase	Asn	0.29%
glutamate 5-kinase	Pro	0.26%
cytidylate kinase	pyrimidines	0.20%
CTP synthase (glutamine)	pyrimidines	0.19%
glucose-1-phosphate adenylyltransferase	glycogen	0.19%
nucleoside-diphosphate kinase	CTP	0.17%
6 reactions in cell wall biosynthesis	peptidoglycans	0.17%
7 reactions in lipopolysaccharide biosynthesis	lipopolysaccharides	0.13%
phospholipid export (2×3 reactions)	phospholipids	0.11%
ATP phosphoribosyltransferase	His, purines	0.11%

Total flux: 69.7 mmol gDW^−1^ h^−1^. Ratios below 0.05% not shown.

Abbreviations: AICAR – 5-aminoimidazole-4-carboxamide ribotide, Arg, Asn, … – biosynthesis of L-amino acids, PRPP – phosphoribosyl pyrophosphate.

The fluxes through the other nucleoside triphosphates are low (GTP: 0.64, UTP: 0.34, CTP: 0.25 mmol gDW^−1^ h^−1^), and their contributions to energy metabolism are not substantial compared to those of ATP and PEP.

#### Phosphoenolpyruvate (PEP)

The split ratios for PEP are shown in Table 3. The total flux through PEP is 16.3 mmol gDW^−1^ h^−1^. As expected, PEP is produced entirely in glycolysis via the enolase reaction. 67.5% of the PEP is used by the phosphotransferase system for glucose import and phosphorylation, yielding pyruvate as the second product. 15.4% is consumed in the anaplerotic reaction catalysed by PEP carboxylase, while 13.0% is used for phosphorylating dihydroxyacetone to dihydroxyacetone phosphate. Another 3.9% is consumed in two reactions in the shikimate pathway, which produces the precursors of the aromatic amino acids. Small quantities of PEP are consumed in the pathways producing peptidoglycans (0.14%) and lipopolysaccharides (0.13%).

**Table 3:** Balance sheet of phosphoenolpyruvate

**Producing fluxes**		
**Reaction/Enzyme**	**Pathway/Product**	**Ratio**
enolase	glycolysis	100%
**Consuming fluxes**		
**Reaction/Enzyme**	**Pathway/Product**	**Ratio**
glucose import (phosphotransferase system)	glycolysis	67.49%
PEP carboxylase	anaplerotic reactions	15.35%
dihydroxyacetone kinase	glycolysis	12.99%
3-phosphoshikimate 1-carboxyvinyltransferase	shikimate pathway	1.95%
3-deoxy-7-phosphoheptulonate synthase	shikimate pathway	1.95%
UDP-N-acetylglucosamine 1-carboxyvinyltransferase	peptidoglycans	0.14%
3-deoxy-8-phosphooctulonate synthase	lipopolysaccharides	0.13%

Total flux: 16.3 mmol gDW^−1^ h^−1^. Ratios below 0.05% not shown.

The large flux through dihydroxyacetone (DHA) kinase is unexpected, as DHA is not known as an intracellular metabolite in *E. coli* except as a usable carbon source [43] or in the degradation of glycerol [44,45], which occurs at a very low rate in this particular scenario (5.5 μmol gDW^−1^ h^−1^, FVA: 4–19 μmol gDW^−1^ h^−1^). In our FBA solution, DHA is produced at a large rate (2.1 mmol gDW^−1^ h^−1^) via fructose-6-phosphate (F6P) aldolase, which cleaves F6P into glyceraldehyde 3-phosphate and DHA. FVA was used to verify that activity in this reaction is not required for optimum growth. If the flux through F6P aldolase is constrained to the condensation direction (i.e. producing F6P from glyceraldehyde 3-phosphate and DHA), all of the glycolytic flux must go through either the fructose bisphosphate aldolase reaction or the sedoheptulose bisphosphate bypass (first described in [46]) in order to achieve optimum growth.

By combining the balance sheets for ATP and PEP, it is possible to compute the net balance of glycolysis: It generates 1.6 molecules of ATP and 1.5 PEP per glucose molecule, while consuming 0.6 ATP and 1.2 PEP, i.e. the balance is positive with a net production of 1.1 ATP and 0.3 PEP per molecule of glucose. This corresponds to 67.4% of the theoretical maximum of 2 molecules of ATP or PEP [47], i.e. one-third of the flux branches off from glycolysis or the pentose phosphate pathway to anabolic pathways producing biomass components.

#### NADH

Equally important to the energy balance of the cell are the reducing equivalents NADH and NADPH. The corresponding balance sheets are shown in Tables 4 and 5. The flux through NADH is 33.7 mmol gDW^−1^ h^−1^. 52.7% is produced in glycolysis, with another 32.1% generated in the formation of acetyl-CoA from pyruvate, while only 8.1% is produced in the TCA cycle. 6.2% of the NADH is generated as a byproduct of amino acid biosynthesis pathways, while the rest is produced as a byproduct of purine and folate metabolism, respectively. Almost all of the NADH is used for energy metabolism: 94.5% enters the electron transport chain via NADH dehydrogenase. Most of the remaining NADH (5.1%) is consumed in fatty acid biosynthesis, while 0.4% is used in the biosynthesis of L-methionine. This is consistent with the general observation that in bacteria grown aerobically on sugars, NADH is produced primarily in the catabolic pathways of glycolysis and TCA cycle and used almost exclusively for ATP generation [47].

**Table 4:** NADH balance

**Producing fluxes**		
**Reaction/Enzyme**	**Pathway/Product**	**Ratio**
glyceraldehyde-3-phosphate dehydrogenase	glycolysis	52.74%
pyruvate dehydrogenase	glycolysis/TCA cycle	32.14%
malate dehydrogenase	TCA cycle	5.35%
phosphoglycerate dehydrogenase	Ser	4.31%
2-oxoglutarate dehydrogenase	TCA cycle	2.78%
3-isopropylmalate dehydrogenase	Leu	1.11%
IMP dehydrogenase	purines	0.60%
histidinol dehydrogenase	His	0.47%
prephenate dehydrogenase	Tyr	0.34%
glycine cleavage system	folate metabolism	0.14%
**Consuming fluxes**		
**Reaction/Enzyme**	**Pathway/Product**	**Ratio**
NADH dehydrogenase	oxidative phosphorylation	94.48%
enoyl-[acyl-carrier-protein] reductase (11 reactions)	fatty acids	5.10%
methylenetetrahydrofolate reductase	Met	0.40%

Total flux: 33.7 mmol gDW^−1^ h^−1^. Ratios below 0.05% not shown.

Abbreviations: His, Leu, … – biosynthesis of L-amino acids.

**Table 5:** NADPH balance

**Producing fluxes**		
**Reaction/Enzyme**	**Pathway/Product**	**Ratio**
glucose 6-phosphate dehydrogenase	pentose phosphate pathway	40.35%
phosphogluconate dehydrogenase	pentose phosphate pathway	40.35%
isocitrate dehydrogenase	TCA cycle	13.40%
methylenetetrahydrofolate dehydrogenase	purines	5.89%
**Consuming fluxes**		
**Reaction/Enzyme**	**Pathway/Product**	**Ratio**
glutamate dehydrogenase	Glu	51.65%
3-oxoacyl-[acyl-carrier-protein] reductase (11 reactions)	fatty acids	13.41%
ketol-acid reductoisomerase (2 reactions)	Val, Leu, Ile, coenzyme A	6.95%
aspartate semialdehyde dehydrogenase	Thr, Met, Lys, peptidoglycans	6.43%
sulfite reductase	sulfur metabolism	4.57%
homoserine dehydrogenase	Thr, Met	4.21%
shikimate dehydrogenase	shikimate pathway	2.28%
dihydrodipicolinate reductase	Lys, peptidoglycans	2.22%
thioredoxin reductase	conversions to other redox carriers	2.14%
N-acetyl-γ-glutamyl-phosphate reductase	Arg, polyamines	2.01%
glutamate 5-semialdehyde dehydrogenase	Pro	1.32%
pyrroline 5-carboxylate reductase	Pro	1.32%
glycerol 3-phosphate dehydrogenase	phospholipids	1.03%
UDP-N-acetylenolpyruvoylglucosamine reductase	peptidoglycans	0.17%
dihydrofolate reductase	folate metabolism	0.16%
dTDP-4-dehydrorhamnose reductase	lipopolysaccharides	0.05%

Total flux: 13.9 mmol gDW^−1^ h^−1^. Ratios below 0.05% not shown.

Abbreviations: Arg, Glu, … – biosynthesis of L-amino acids.

#### NADPH

At 13.9 mmol gDW^−1^ h^−1^, the flux through NADPH is 59% lower than that through NADH. The largest part (80.7%) stems from the oxidative pentose phosphate pathway (PPP). Another 13.4% is produced by the enzyme isocitrate dehydrogenase (ICD) in the TCA cycle, while 5.9% is generated as a byproduct in purine metabolism. It has been shown in ^13^C tracer experiments that in *E. coli* grown aerobically on glucose, 35–45% of the NADPH is produced from NADH and NADP^+^ by the membrane-bound proton-translocating transhydrogenase PntAB, while another 35–45% is produced in the PPP, and 20–25% is produced by ICD [48]. In contrast, FBA predicted no transhydrogenase activity for the model, and FVA confirmed that under the constraint of maximum biomass production, no significant transhydrogenase flux is possible. The prediction that malic enzyme does not play a significant role for NADPH generation in the scenario under consideration (confirmed by FVA) is in agreement with the experimental observations [48].

An exploratory FBA in an alternative scenario with a forced transhydrogenase flux of 5 mmol gDW^−1^ h^−1^ yielded a flux distribution in agreement with the results of [48], predicting 36% of the NADPH to be produced by transhydrogenase, 21% by ICD, and 37% by the PPP. At the same time, this FBA solution was slightly better at predicting the oxygen uptake rate (16.7 vs. 16.5 mmol gDW^−1^ h^−1^; experimental value: 18.2 ± 0.8 mmol gDW^−1^ h^−1^[49]) than that obtained with the original parameters, while predicting roughly the same values for growth rate and CO_2_ emission rate.

Apart from being a biomass component itself, NADPH is consumed in 33 reactions with a flux above 0.1 μmol gDW^−1^ h^−1^. 77.8% is used for the biosynthesis of various amino acids, of which two-thirds (51.7% of the total NADPH) is accounted for by L-glutamate alone, followed by L-threonine (6.5%) and L-lysine (4.1%). The biosyntheses of all three branched-chain amino acids together consume 7.0% of the NADPH. Other major processes consuming NADPH are fatty acid biosynthesis (13.4%) and sulfur assimilation (4.6%). 2.1% is used for restoring thioredoxin to the reduced state, which in turn is used for sulfur assimilation (71%) and biosynthesis of deoxyribonucleotides (29%). 1.0% of the NADPH is used in phospholipid biosynthesis, while the remainder is consumed in the biosynthesis of other biomass components, including polyamines, peptidoglycans, lipopolysaccharides, and various cofactors. 0.16% is consumed in folate metabolism. Both the lower flux through NADPH compared to NADH and the distribution of the NADPH-consuming flux over a large number of reactions in various biosynthetic pathways are in good agreement with the paradigm that while NADH is used for energy metabolism, NADPH acts, among other functions, as a reducing agent in anabolic pathways [47].

### Selected branch points

A detailed analysis of the split ratios in selected metabolite nodes was performed with the two goals of elucidating the metabolic roles of metabolite species and identifying the relative contributions of biochemical pathways to biomass formation and energy generation, respectively, in the scenario under consideration.

Table 6 shows the fluxes producing and consuming **2-oxoglutarate**. The total flux through the metabolite is 8.1 mmol gDW^−1^ h^−1^, of which 77.0% is generated as a byproduct of various transaminases, which transfer the amino group of L-glutamate to different acceptors, producing L-amino acids. In contrast, only 23.0% is produced *de novo* from isocitrate in the TCA cycle. The consuming side also reflects the presence of a transamination cycle: 88.5% of the 2-oxoglutarate is converted to L-glutamate, while only 11.5% is metabolised in the TCA cycle.

**Table 6:** Split ratios of 2-oxoglutarate

**Producing fluxes**		
**Reaction/Enzyme**	**Pathway/Product**	**Ratio**
aspartate transaminase	Asp	29.99%
isocitrate dehydrogenase	TCA cycle	22.96%
phosphoserine transaminase	Ser	17.85%
L-alanine transaminase	Ala	5.78%
leucine transaminase	Leu	4.61%
valine transaminase	Val	4.33%
succinyldiaminopimelate transaminase	Lys, peptidoglycans	3.80%
acetylornithine transaminase	Arg, polyamines	3.45%
isoleucine transaminase	Ile	2.97%
phenylalanine transaminase	Phe	1.89%
tyrosine transaminase	Tyr	1.41%
histidinol-phosphate transaminase	His	0.97%
**Consuming fluxes**		
**Reaction/Enzyme**	**Pathway/Product**	**Ratio**
glutamate dehydrogenase	Glu	88.48%
2-oxoglutarate dehydrogenase	TCA cycle	11.51%

Total flux: 8.12 mmol gDW^−1^ h^−1^. Ratios below 0.05% not shown.

Abbreviations: Ala, Arg, … – biosynthesis of L-amino acids.

Subtracting the flux of 6.3 mmol gDW^−1^ h^−1^ going through the transamination cycle yields a net balance, in which 100% of the 2-oxoglutarate (1.9 mmol gDW^−1^ h^−1^) is produced in the TCA cycle. Of this non-cyclic flux, 50.1% continues in the TCA cycle, while 49.8% is converted to L-glutamate as starting molecule of various biosynthetic pathways.

A second transamination cycle exists between L-glutamine as amino-group donor and **L-glutamate**. The balance sheet for L-glutamate (Table 7) shows both transamination cycles: Of the total flux of 8.5 mmol gDW^−1^ h^−1^ going through L-glutamate, 85.0% is produced from 2-oxoglutarate, while 15.0% is the result of transamination with L-glutamine as amino-group donor. 74.0% is used for transferring amino groups, while 17.6% is converted to L-glutamine.

**Table 7:** Split ratios of L-glutamate

**Producing fluxes**		
**Reaction/Enzyme**	**Pathway/Product**	**Ratio**
glutamate dehydrogenase	TCA cycle	85.02%
amidophosphoribosyltransferase	purines	4.40%
phosphoribosylformylglycinamidine synthase	purines	4.40%
GMP synthase	purines	2.39%
CTP synthase	CTP	1.57%
imidazole-glycerol-3-phosphate synthase	His, purines	0.93%
glutamine-fructose-6-phosphate transaminase	peptidoglycans, lipopolysaccharides	0.72%
anthranilate synthase	Trp	0.56%
**Consuming fluxes**		
**Reaction/Enzyme**	**Pathway/Product**	**Ratio**
aspartate transaminase	Asp	28.82%
glutamine synthetase	Gln	17.57%
phosphoserine transaminase	Ser	17.15%
L-alanine transaminase	Ala	5.55%
leucine transaminase	Leu	4.43%
valine transaminase	Val	4.16%
succinyldiaminopimelate transaminase	Lys, peptidoglycans	3.65%
N-acetylglutamate synthase	Arg, polyamines	3.31%
acetylornithine transaminase	Arg, polyamines	3.31%
isoleucine transaminase	Ile	2.85%
Biomass		2.59%
glutamate 5-kinase	Pro	2.17%
phenylalanine transaminase	Phe	1.82%
tyrosine transaminase	Tyr	1.36%
histidinol-phosphate transaminase	His	0.93%
glutamate racemase	peptidoglycans	0.28%
glutamyl-tRNA synthetase	cobalamin, heme	0.05%

Total flux: 8.46 mmol gDW^−1^ h^−1^. Ratios below 0.05% not shown.

Abbreviations: Ala, Arg, … – biosynthesis of L-amino acids.

The net balance of L-glutamate with all fluxes in the two transamination cycles removed is shown in Table 8. The remaining 0.93 mmol gDW^−1^ h^−1^, which are produced exclusively from 2-oxoglutarate, are chiefly used for the biosynthesis of the amino acids L-arginine (26.4%), L-glutamine (23.5%), and L-proline (19.8%), while another 23.5% enters the biomass directly. The rest is consumed in the biosynthesis of polyamines (3.7%), peptidoglycans (2.5%), and various cofactors.

**Table 8:** Split ratios of L-glutamate discounting transamination cycles

**Producing fluxes**		
**Reaction**	**Pathway**	**Ratio**
glutamate dehydrogenase	TCA cycle	100%
**Consuming fluxes**		
**Reaction**	**Pathway**	**Ratio**
N-acetylglutamate synthase	Arg, polyamines	30.10%
glutamine synthetase	Gln	23.51%
Biomass		23.51%
glutamate 5-kinase	Pro	19.75%
glutamate racemase	peptidoglycans	2.54%
glutamyl-tRNA synthetase	cobalamin, heme	0.49%
dihydrofolate synthase	folate metabolism	0.08%

Total flux: 0.93 mmol gDW^−1^ h^−1^. Ratios below 0.05% not shown.

Abbreviations: Arg, Gln, Pro – biosynthesis of L-amino acids.

**Figure 1:**
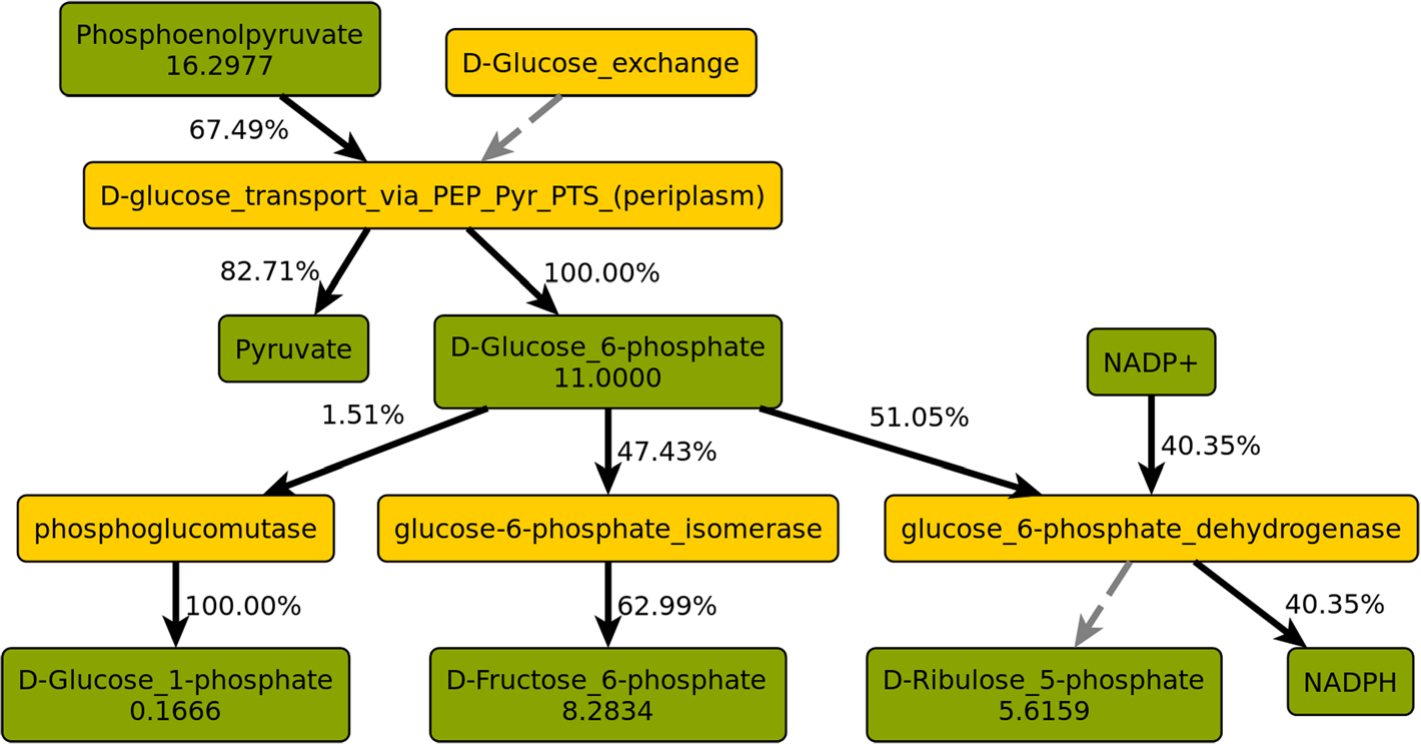
**Split ratios of glucose 6-phosphate and adjacent nodes.** Green: Metabolite nodes, yellow: enzyme nodes. Numbers in metabolite nodes are total flux in mmol gDW^−1^ h^−1^. Edge labels are split ratios as fractions of the flux through the adjacent metabolite node.

**Glucose 6-phosphate (G6P)** is the first branch point in glycolysis. It is shown in metabolic context (two levels of depth) with split ratios in Figure 1. All G6P is generated from glucose via the phosphotransferase system, which is both the main consumer of PEP and the main producer of pyruvate. This illustrates how, in the scenario under consideration, the phosphotransferase system effectively couples the first step of glycolysis (glucose uptake and phosphorylation) with the last step (dephosphorylation of PEP to pyruvate) [50]. Only 47.4% of the G6P continues along the glycolytic path, while 51.1% enters the pentose phosphate pathway (PPP). The remaining 1.5% is converted to glucose 1-phosphate, of which 79.1% is used to build glycogen, while the rest is consumed in several reactions of lipopolysaccharide biosynthesis. It should be noted that much of the carbon that initially enters the PPP returns to the glycolytic route via transaldolase and transketolase, respectively. Of the 66.0 mmol gDW^−1^ h^−1^ flux of carbon atoms through G6P, 48.9 mmol gDW^−1^ h^−1^ (74.1%) reach PEP via glycolysis. The fluxes in the whole PPP and its connections with glycolysis are shown in Additional file 1: Figure S1.

**Figure 2:**
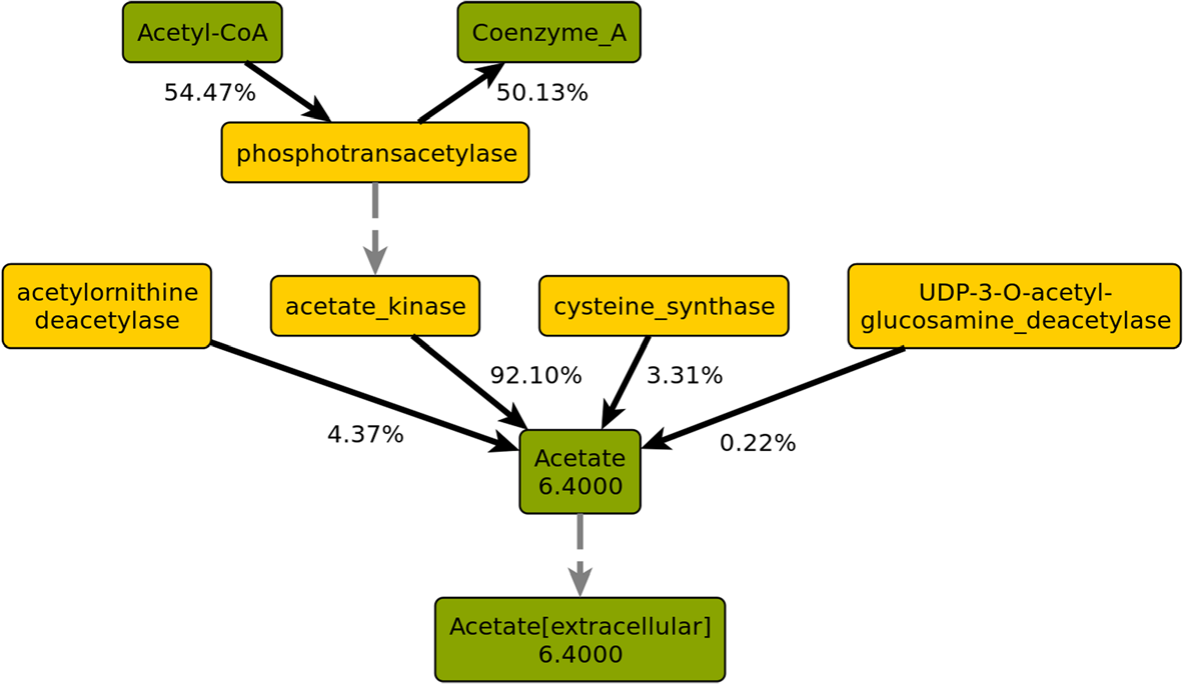
**Split ratios of acetate and adjacent nodes.** Green: Metabolite nodes, yellow: enzyme nodes. Numbers in metabolite nodes are total flux in mmol gDW^−1^ h^−1^. Edge labels are split ratios as fractions of the flux through the adjacent metabolite node.

Figure 2 shows the split ratios of **acetate** in its metabolic context. Acetate is produced and secreted at the forced rate of 6.4 mmol gDW^−1^ h^−1^. While some acetate is produced as a byproduct of arginine metabolism (4.4%), L-cysteine biosynthesis (3.3%), and the biosynthesis of lipopolysaccharides (0.2%), the largest part (92.1%) is produced from acetyl-CoA via phosphotransacetylase and acetate kinase. At this rate of acetate secretion, more than 50% of all acetyl-CoA takes the acetate-exporting route instead of the TCA cycle. FVA was used to verify that indeed the forced acetate secretion is the sole reason for activity in this route: If this constraint is disabled, acetate kinase and phosphotransacetylase work in the opposite direction to salvage the acetate produced as a byproduct of the other pathways. FVA also confirmed that this route is the cheapest way with regard to biomass yield to produce acetate at the required rate.

### Metabolic cycles and metabolite pools

**Figure 3:**
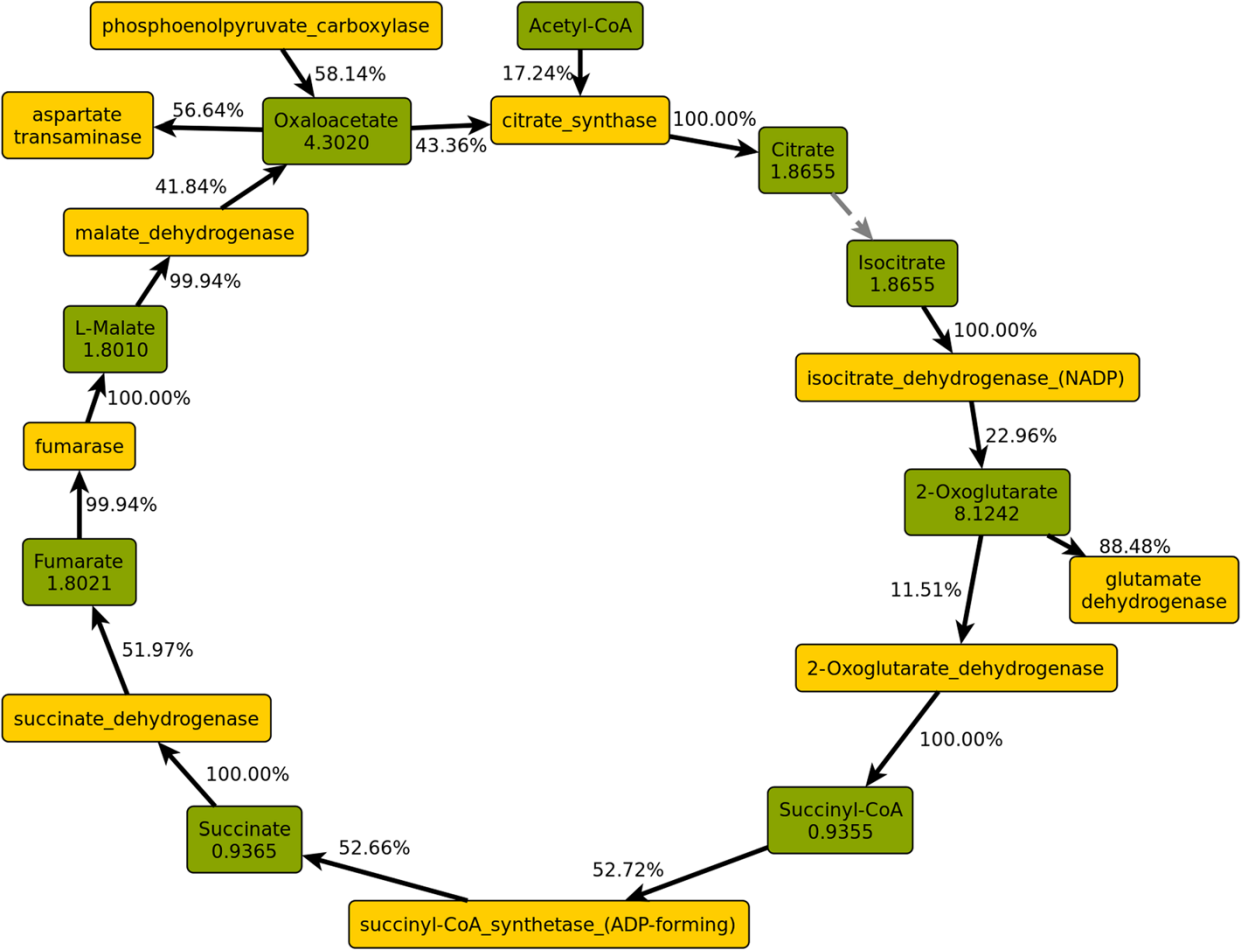
**Metabolic fluxes and split ratios in the TCA cycle.** Green: Metabolite nodes, yellow: enzyme nodes. Numbers in metabolite nodes are total flux in mmol gDW^−1^ h^−1^. Edge labels are split ratios as fractions of the flux through the adjacent metabolite node.

Branch point analysis permits the visualisation of whole pathways with actual fluxes. This is particularly useful for metabolic cycles and metabolite pools that are depleted and replenished via many different reactions. Figure 3 shows all fluxes in the TCA cycle in the scenario under consideration.

As mentioned above, only about one-sixth of the acetyl-CoA enters the TCA cycle, while the majority is converted to acetate for secretion. All the flux entering citrate synthase also goes through isocitrate dehydrogenase, i.e. all the citrate is converted to 2-oxoglutarate, which is the first of two major branch points towards anabolic pathways. As described above, only 50.1% of the 2-oxoglutarate continues in the TCA cycle, forming succinyl-CoA. All of the succinyl-CoA is eventually converted to succinate, 52.7% within the TCA cycle, 33.0% in L-lysine biosynthesis, and 14.3% in the biosynthesis of L-methionine. All of the succinate is then oxidized to fumarate, but succinate dehydrogenase is not the sole source of fumarate. Virtually all of the fumarate is converted to oxaloacetate via fumarase and malate dehydrogenase, but the largest part of the oxaloacetate (58.1%) results from the anaplerotic reaction catalysed by PEP carboxylase.

**Figure 4:**
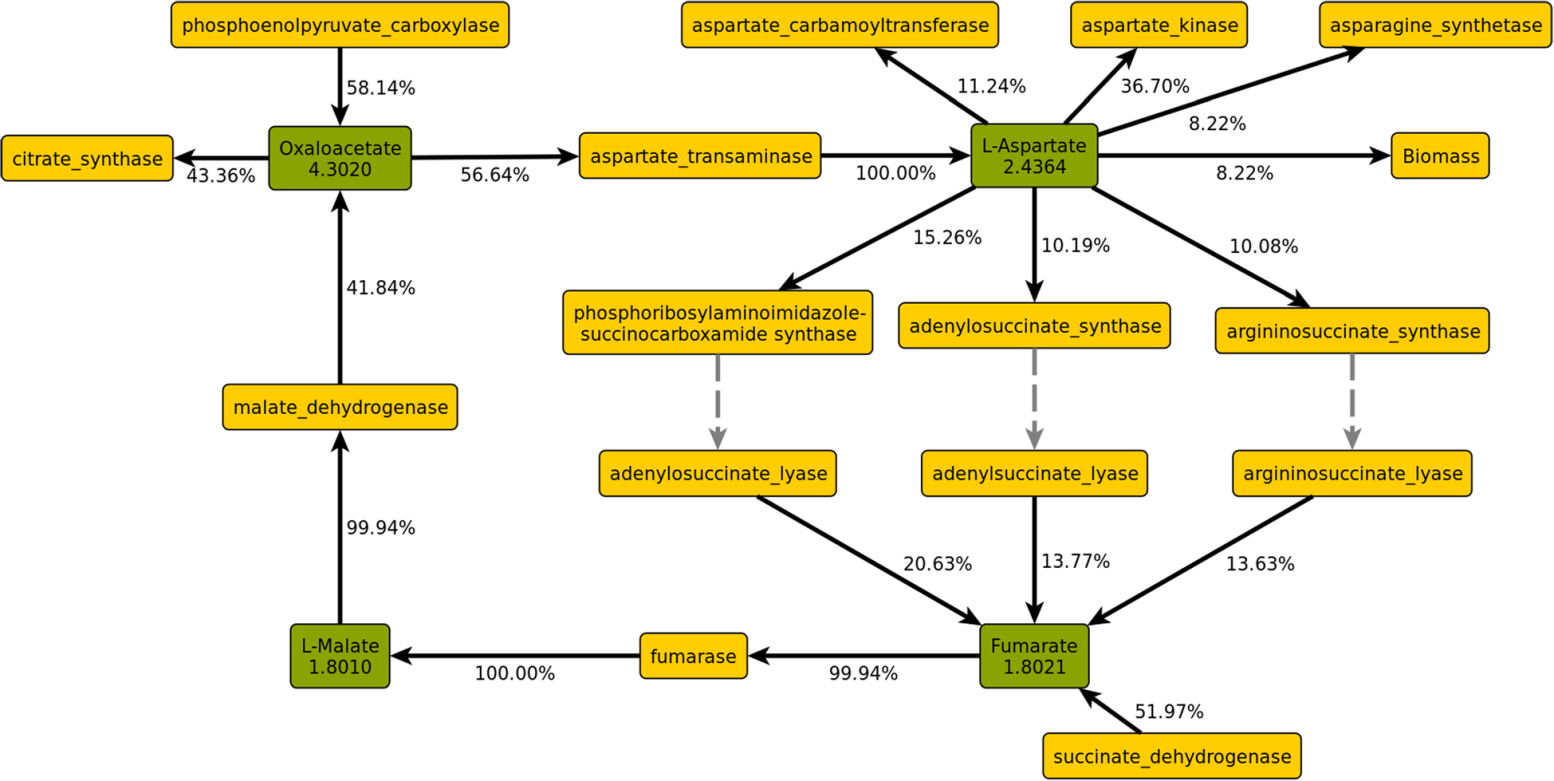
**Cycle between oxaloacetate and fumarate.** Green: Metabolite nodes, yellow: enzyme nodes. Numbers in metabolite nodes are total flux in mmol gDW^−1^ h^−1^. Edge labels are split ratios as fractions of the flux through the adjacent metabolite node.

Oxaloacetate is the second major branch point of the TCA cycle. 56.6% of the flux through this node is redirected towards anabolism, forming L-aspartate as the first step. However, branch point analysis revealed the presence of a cycle between oxaloacetate and fumarate (shown in Figure 4). In this cycle, which carries a flux of 0.87 mmol gDW^−1^ h^−1^, L-aspartate formed from oxaloacetate transfers its amino group to different acceptors in purine and arginine metabolism, respectively, yielding fumarate, which is converted back to oxaloacetate in the TCA cycle. Correcting for this cycle yields a ratio of 54.3% of oxaloacetate remaining in the cycle and 45.7% entering anabolic pathways. The fraction of anaplerotic oxaloacetate (produced by PEP carboxylase) increases to 72.8% with this correction.

While the predicted fraction of acetyl-CoA converted to acetate (54%) is close to the value determined by Fischer et al. [49] (55–59%), the fraction predicted to enter the TCA cycle is considerably lower (17% vs. 25–30%). The measured fluxes in the TCA cycle are almost twice as high as the predicted ones – for instance, the flux through isocitrate dehydrogenase was measured to be 27–33% of the glucose uptake rate, while the predicted value is 17%.

In the alternative scenario with a forced transhydrogenase flux of 5 mmol gDW^−1^ h^−1^, the flux through the TCA cycle is considerably larger (isocitrate dehydrogenase: 2.9 vs. 1.9 mmol gDW^−1^ h^−1^), which is much closer to experimental observations – 2.9 mmol gDW^−1^ h^−1^ amounts to 26% of the glucose uptake rate of 11 mmol gDW^−1^ h^−1^. As a result, the fraction of NADH produced via the TCA cycle increases from 8.1% to 12.9%.

**Figure 5:**
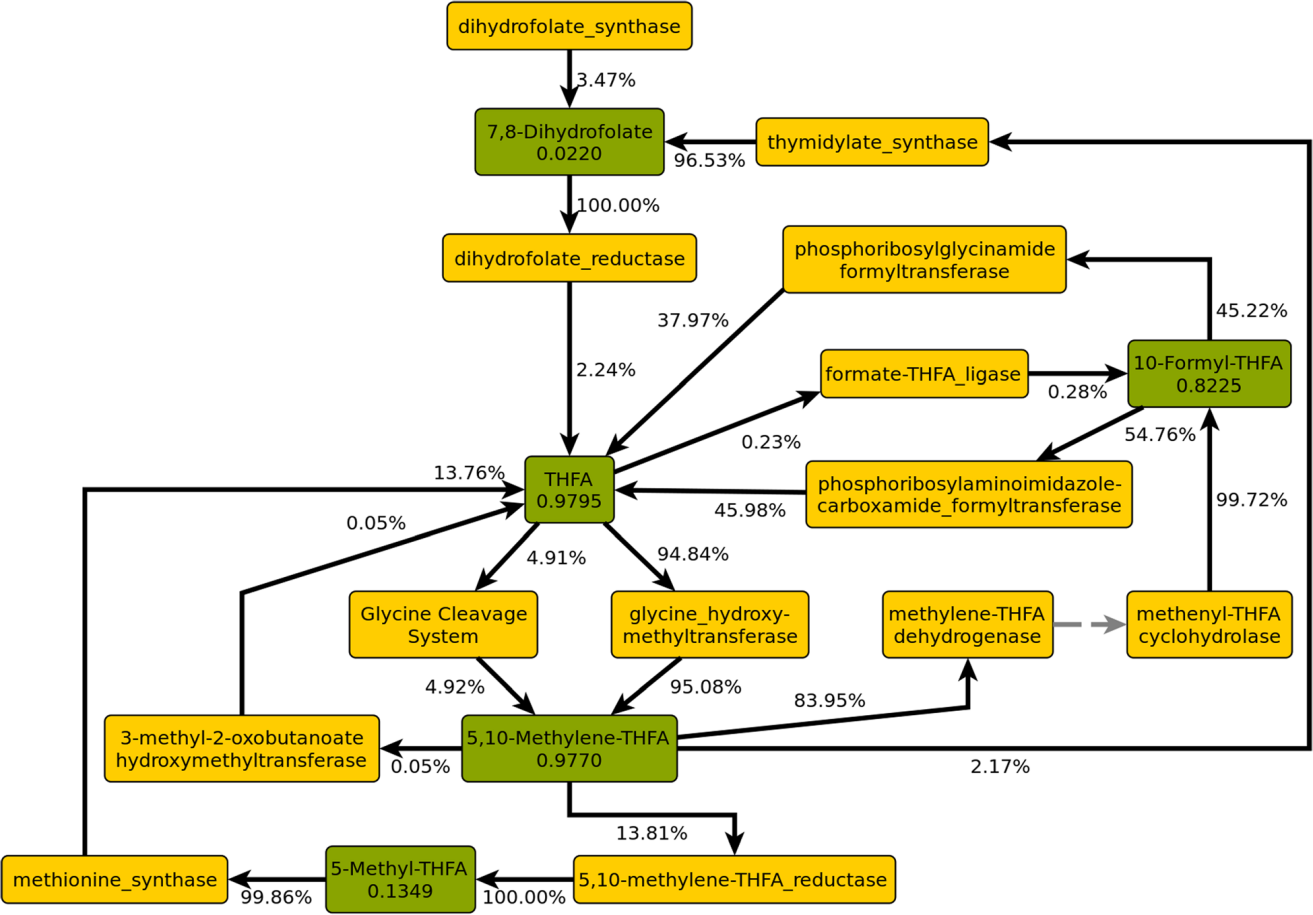
**Metabolite fluxes and split ratios within the tetrahydrofolate pool.** Green: Metabolite nodes, yellow: enzyme nodes. Numbers in metabolite nodes are total flux in mmol gDW^−1^ h^−1^. Edge labels are split ratios as fractions of the flux through the adjacent metabolite node.

5,6,7,8-Tetrahydrofolate (THFA), via its activated forms 5,10-methylene-THFA, 5-methyl-THFA, and 10-formyl-THFA, is one of the cell’s most important donors of one-carbon groups. In addition, it can act as a reducing agent. Figure 5 shows all fluxes within the folate-dependent one-carbon pool.

The total flux through THFA is 0.98 mmol gDW^−1^ h^−1^, but only 0.08% of this stems from *de-novo* synthesis, while most of the THFA circulates. Almost all of the THFA is converted to 5,10-methylene-THFA, where the transferred methylene group stems from cleavage of either L-serine (94.8%) or glycine (4.9%). 0.23% is converted to 10-formyl-THFA instead, reassimilating formate at the cost of ATP hydrolysis. The largest part of the 5,10-methylene-THFA (84.0%) is also subsequently converted to 10-formyl-THFA, which transfers a formyl group in two reactions in the biosynthesis of purine nucleotides. 13.8% of the 5,10-methylene-THFA is converted to 5-methyl-THFA, which donates a methyl group in the biosynthesis of L-methionine. Another 2.2% is consumed in the biosynthesis of dTMP, where it donates both a methylene group and two electrons, resulting in dihydrofolate as the depleted form, which is subsequently reduced back to THFA. Finally, 5,10-methylene-THFA transfers a methylene group in the first step of coenzyme A biosynthesis, which accounts for 0.05% of the total flux.

### Essentiality of metabolite nodes

548 of the 557 metabolite nodes carrying a flux (98%) were identified as essential for optimal growth by metabolite flux minimisation. The remaining nine metabolites are shown in Table 9, along with the number of reactions in which they occur. As predicted by the model, they are intermediates in nonessential pathways. The results for the intracellular metabolites agree with the findings of Kim et al. based on the earlier model iAF1260 [32]. However, that study did not consider periplasmic or extracellular metabolites. Menaquinone-8, which is involved in twenty-three reactions, was identified as the most promiscuous nonessential metabolite. While menaquinone-8 does not carry a flux in the particular FBA solution used in this work, FVA revealed that there are reactions involving this metabolite that can carry a flux under the constraint of optimal biomass production. Of the ten reactions involving thioredoxin, seven can actually carry a flux, as determined by FVA.

**Table 9:** Metabolites carrying a flux but predicted to be nonessential

**Metabolite**	**Number of reactions**
adenosine	7
α-D-ribose 1-phosphate	7
fructose 1,6-bisphosphate	4
glycine[periplasm]	4
hydroxypyruvate	3
H+[extracellular]	6
thioredoxin (reduced)	10
thioredoxin (oxidized)	10
[cell wall precursor of two linked disaccharide pentapeptide and tetrapeptide murein units]	3

Shown is the number of reactions in which each nonessential metabolite appears in the network.

Adenosine and α-D-ribose 1-phosphate occur in a salvage pathway for adenine, which can alternatively be converted to AMP in one step via adenine phosphoribosyltransferase. Fructose 1,6-bisphosphate can be completely bypassed in the model by the sedoheptulose bisphosphate bypass, where phosphofructokinase and fructose bisphosphate aldolase act on sedoheptulose 7-phosphate from the pentose phosphate pathway and sedoheptulose 1,7-bisphosphate instead of fructose 6-phosphate and fructose 1,6-bisphosphate, respectively. Since these pathways are stoichiometrically equivalent, the use of either pathway allows optimal biomass production. Periplasmic glycine is an intermediate in a transport cascade that has the net effect of importing Ca^2+^ ions. As the only Ca^2+^ importer available in the model is a Ca^2+^/Na^+^ antiporter, a second transporter must be active to pump Na^+^ ions to the cytosol. A large number of Na^+^ symporters (including for glycine, glycolate, and acetate) are present in the model, and most of these have the same energy cost, as most of the used organic compounds can be exported to the periplasm via 1:1 H^+^ symport. Excess intracellular protons are exported to the extracellular space in our FBA solution. There exists a stoichiometric alternative via a periplasmic ferroxidase, which produces water from protons, oxygen, and electrons donated by Fe^2+^, so that no protons are exported to the medium. The additional import flux of Fe^2+^ required for the conversion of all excess protons in this way is 14.0 mmol gDW^−1^ h^−1^, which is equal to the required Fe^3+^ export flux. Hydroxypyruvate occurs as an intermediate in the reaction pair hydroxypyruvate isomerase + hydroxypyruvate reductase, which is stoichiometrically equivalent to the single reaction catalysed by tartronate semialdehyde reductase. Thioredoxin can be replaced by other reducing agents, as the model contains alternative reactions to those catalysed by thioredoxin-dependent enzymes.

The result that most active metabolite nodes are essential is complemented by the observation that according to FVA, only 76 of the 552 active reactions (14%) are nonessential, i.e. most of the active reactions need to carry a flux for optimal biomass production.

Table 10 shows the thirty-two intracellular metabolites with the largest minimum turnover rates. Many entries in this list are ubiquitous metabolites, such as H^+^, H_2_O, and phosphate, or circulating currency metabolites or cofactors, such as ATP/ADP, NADH/NAD^+^, and coenzyme A. The list also contains a large number of glycolytic metabolites, which is due to the high flux through glycolysis, as all the carbon flux initially passes through glycolysis and branches off to other pathways only gradually. Metabolite flux minimisation also predicts large required fluxes through O_2_ and CO_2_, which is expected for the aerobic condition. Due to the transamination cycle described above, L-glutamate and 2-oxoglutarate have high minimum fluxes. Acetate and acetyl phosphate appear in the list due to the high forced acetate export flux. In addition, metabolites from the pentose phosphate pathway have to carry a high flux, as that constitutes the cheapest way for the model to produce NADPH.

**Table 10:** Intracellular metabolites with the largest minimum fluxes

**Metabolite**	**Minimum flux**	**Metabolite**	**Minimum flux**
H+	239.2	NADP+	13.9
H2O	108.1	NADPH	13.9
ADP	69.7	pyruvate	13.3
ATP	69.7	coenzyme A	11.8
phosphate	66.9	glucose 6-phosphate	11.0
NAD+	33.7	acetyl-CoA	10.8
NADH	33.7	ammonium	9.4
ubiquinol-8	33.0	L-glutamate	8.5
ubiquinone-8	33.0	dihydroxyacetone phosphate	8.2
CO2	23.5	2-oxoglutarate	8.1
glyceraldehyde 3-phosphate	17.8	fructose 6-phosphate	6.6
3-phospho-D-glycerate	17.7	acetate	6.4
1,3-bisphospho-D-glycerate	17.7	acetyl phosphate	5.9
O2	16.5	6-phospho-D-gluconate	5.6
2-phospho-D-glycerate	16.3	6-phospho-D-glucono-1,5-lactone	5.6
PEP	16.3	D-ribulose 5-phosphate	5.6

Shown is the minimum flux (in mmol gDW^−1^ h^−1^) that each metabolite has to carry for the growth rate to be maximal.

For carbon-containing metabolites, a more useful ranking is not by total flux, but by carbon flux, i.e. the product of metabolite flux and number of carbon atoms in the compound. The twenty intracellular non-cofactor metabolites with the highest required carbon fluxes are shown in Table 11. Half of these occur in glycolysis, and another five are intermediates of the pentose phosphate pathway. The remaining five metabolites in the list are L-glutamate, 2-oxoglutarate, oxaloacetate (all involved in transamination cycles), CO_2_, and acetate. The example of glucose 6-phosphate illustrates the usefulness of this alternative flux measure: It is only ranked eighth among the carbon metabolites in Table 10 but is the most active metabolite by carbon flux.

**Table 11:** Metabolites with the largest essential carbon fluxes

**Metabolite**	**C atoms**	**Minimum flux**	**Minimum C flux**
glucose 6-phosphate	6	11	66
glyceraldehyde 3-phosphate	3	17.75	53.26
3-phospho-D-glycerate	3	17.75	53.24
1,3-bisphospho-D-glycerate	3	17.75	53.24
2-phospho-D-glycerate	3	16.3	48.89
PEP	3	16.3	48.89
L-glutamate	5	8.46	42.28
2-oxoglutarate	5	8.12	40.62
pyruvate	3	13.3	39.9
fructose 6-phosphate	6	6.59	39.55
6-phospho-D-gluconate	6	5.62	33.7
6-phospho-D-glucono-1,5-lactone	6	5.62	33.7
D-ribulose 5-phosphate	5	5.62	28.08
dihydroxyacetone phosphate	3	8.23	24.68
CO2	1	23.5	23.5
acetyl-CoA	2^*^	10.82	21.64
oxaloacetate	4	4.3	17.21
D-xylulose 5-phosphate	5	3.09	15.47
acetate	2	6.4	12.8
D-ribose 5-phosphate	5	2.5	12.5

Fluxes are in mmol gDW^−1^ h^−1^. ^*^discounting CoA moiety.

## Discussion

We have demonstrated how the energy metabolism of the cell, as predicted by the model, can be characterised by compiling the balance sheets of a handful of currency metabolites. The split ratios of currency metabolites can be used to check a metabolic model for biological plausibility even in the absence of specific experimental data for the studied organism, as energy metabolism tends to follow certain well-studied paradigms solely based on ecology (phototrophic vs. chemotrophic, aerobic vs. anaerobic, etc.). Thus, split-ratio analysis is a powerful tool for plausibility checking and refinement of metabolic models.

By inspecting selected metabolites in detail, we have demonstrated how branch point analysis can be used to elucidate the metabolic roles of compounds occurring in the network in the scenario under consideration. For instance, branch point analysis can be used to determine whether a metabolite carrying a flux acts as a mere pathway intermediate, is produced solely as a biomass component, acts as energy or redox carrier, acts as a group donor, or occurs as a mere byproduct, which is recycled to central metabolism.

Split ratio analysis can also be used in an exploratory approach, as the relevant context for interpreting the flux through any reaction is provided by its split ratios with respect to the flux sums in each of the substrates and products involved. Branch point analysis presents just this context at a glance. To revisit the questions posed in the Background section: The flux through glutamate dehydrogenase is easily interpretable when expressed as a fraction of the total producing glutamate flux, the total consuming 2-oxoglutarate flux, the total consuming NADPH flux, and possibly the total ammonia-consuming flux. Likewise, the flux through aspartate transaminase is best expressed as a fraction of the total fluxes through oxaloacetate, aspartate, glutamate, and possibly 2-oxoglutarate.

Key split ratios, such as those of 2-oxoglutarate and oxaloacetate, can be used to identify the relative contributions of a pathway to energy generation and biomass production, respectively. Hence, a metabolite-centric view on flux distributions can guide metabolic engineering, where the goal is often to optimise split ratios between pathways leading to product formation, biomass production, and energy generation in order to achieve maximum product yield [51,52]. In fact, if enzymes in a pathway producing a desired product are overexpressed, the effect is not only an increased flux through this pathway, but also a shift in split ratios, as all matter entering the product-forming route is diverted from energy and growth metabolism.

It should be noted that only a small fraction of the metabolites in the network are branch points in any given scenario, so that the global network behaviour can be characterised via a small number of detailed analyses.

By reducing the metabolic network to only those nodes that are branch points, branch point analysis allows the visualisation of all fluxes within a metabolic cycle, a metabolite pool, or even larger subsystems, as we have demonstrated using the examples of the TCA cycle, the folate-dependent one-carbon pool, and the superpathway consisting of glycolysis and the pentose phosphate pathway. Using advanced graph layouting algorithms (possibly guided by a manually drawn pathway map), it may even be possible to produce a clear and informative visualisation of the steady-state fluxes in the whole network. In addition, branch point analysis allows the graphical identification of metabolic cycles, which can subsequently be analysed for their biological significance.

The system-wide analysis of branch points and split ratios is complemented by metabolite essentiality analysis, implemented using either flux-sum analysis or metabolite flux minimisation. It should be noted that this type of analysis is only possible within a metabolite-centric view. For instance, merely examining the fluxes or flux variabilities of the twenty-three reactions in the *E. coli* model involving menaquinone-8 or the ten reactions involving thioredoxin cannot reveal these metabolites to be nonessential for optimal growth. In the case of thioredoxin, seven of the ten fluxes can actually carry a flux under the constraint of optimal biomass production.

In metabolite flux minimisation, a nonessential metabolite is identified as a metabolite with a minimum flux sum of zero under the constraint of optimal biomass flux. In flux-sum analysis, in contrast, a nonessential metabolite is one that can be removed from the network (modelled by restricting the fluxes consuming the metabolite to zero) without a decrease in biomass flux [32]. The two definitions are obviously equivalent. However, as metabolite flux minimisation only alters the objective function and not the constraints defining the solution space, it is amenable to the same strategy that is used for speeding up FVA [53] and, thus, is more computationally efficient.

Applying the metabolite-centric view introduced here to the published model iJO1366 of *E. coli*, we discovered a number of hitherto not discussed peculiarities in network behaviour:

A systematic analysis of the balance sheets of all energy metabolites revealed unexpectedly strong activity of dihydroxyacetone kinase, which further led to the discovery of the high activity of fructose-6-phosphate (F6P) aldolase in the FBA solution. Usually, F6P is phosphorylated once more to fructose 1,6-bisphosphate, and only that is cleaved (into glyceraldehyde 3-phosphate and dihydroxyacetone phosphate). F6P aldolase was discovered in *E. coli* in 2000 [54], but its function *in vivo* remains obscure. Moreover, nothing is known about *in-vivo* expression of this enzyme. Schürmann and Sprenger [54] note that the F6P cleavage reaction is 10 kJ mol^−1^ more endergonic than the fructose-1,6-bisphosphate cleavage reaction, making it unlikely for the reaction to proceed in the cleavage direction rather than the reverse direction. The central role, tight regulation, and ubiquitous presence of fructose-bisphosphate aldolase in the glycolytic pathways of all organisms capable of classical (Embden-Meyerhof-Parnas) glycolysis sheds further doubt on a notable alternative being active in *E. coli*. While the two reaction pairs F6P kinase + fructose-bisphosphate aldolase and F6P aldolase + dihydroxyacetone kinase are stoichiometrically equivalent except for the phosphorylating agent involved (ATP vs. PEP), the model would likely reflect the metabolic behaviour *in vivo* more accurately if the flux through F6P aldolase were constrained so as to disallow the cleavage direction. Possibly, the reaction should even be omitted altogether until more is known about *in-vivo* expression and regulation of the enzyme. The inclusion of F6P aldolase in the model (which is not discussed by the authors of iJO1366 or either of its predecessors iAF1260 and iJR904) may represent a case of a spurious enzyme function, possibly resulting from blanket inclusion of all enzymes found in an automated database search. If added with unconstrained flux, such cryptic enzymes can dramatically lower the quality of the predictions made by the model.

A systematic survey of metabolite essentiality resulted in the equally surprising observation that in the model, fructose 1,6-bisphosphate is nonessential for optimal growth. The reason was found in the sedoheptulose bisphosphate bypass, which is equivalent to the standard reactions of phosphofructokinase and fructose bisphosphate aldolase with regard to biomass flux. Notable activity in this pathway has, however, only been observed in transaldolase-deficient mutants, but never in wild-type strains of *E. coli*[46]. It is likely that the affinities of these enzymes to the respective sedoheptulose phosphates are much lower than to the corresponding fructose phosphates, so that *in vivo*, the bypass reactions would not be expected to display significant activity unless there is an accumulation of sedoheptulose 7-phosphate for some reason. Therefore, it would be advisable to constrain the fluxes through the bypass reactions to zero when specifically studying the wild-type network.

We found a strong discrepancy between the predicted NADPH balance and that measured in [48]. This discrepancy is explained by the fact that *in vivo*, a more costly reaction (proton-translocating transhydrogenase) accounts for a substantial fraction of the NADPH produced. Flux through this reaction reduces the number of protons in the periplasm available to ATP synthase, which means that more carbon has to be respired to CO_2_, lowering the biomass yield. Therefore, this reaction is not used for the generation of NADPH in an FBA flux distribution optimised for biomass production. To improve the capability of the model to predict fluxes in ‘aerobic growth on sugars’ scenarios, a flux through this reaction needs to be enforced, as we could show in preliminary tests with altered simulation parameters. Ideally, this should be done by postulating a stoichiometric coupling to one or more other reactions instead of fixing the flux at an absolute value. For instance, a constant ratio of periplasmic protons might be forced to take this route, resulting in a stoichiometric coupling to ATP synthase. Or, if that should reproduce experimental results more faithfully, the flux through this reaction could be coupled to the fluxes through other NADPH-producing reactions. In the absence of new experimental data, a black-box approach could also be taken, where the transhydrogenase flux would be set to a fixed fraction of the carbon flux entering the system. Of course, the ratio between NADPH-producing reactions may change dramatically depending on carbon source and availability of oxygen, so that any such constraints are only valid for the particular scenario of carbon-limited aerobic growth on sugars.

In the original scenario, the predicted flux going through the TCA cycle (~ 1.9 mmol gDW^−1^ h^−1^) is very low in comparison to those through acetyl-CoA (10.8 mmol gDW^−1^ h^−1^) and PEP (16.3 mmol gDW^−1^ h^−1^). In addition, the anabolic fluxes branching from the TCA cycle are very large (50.1% of 2-oxoglutarate and 45.7% of oxaloacetate). Taken together, these results indicate that in the original scenario, the primary purpose of the TCA cycle, as predicted by the model, is not energy generation but production of C_5_ and C_4_ compounds for the biosynthesis of biomass components. This can also be seen in the low contribution of the TCA cycle to NADH and NADPH production. When adding a flux through proton-translocating transhydrogenase of 5 mmol gDW^−1^ h^−1^, the role of the TCA cycle shifts somewhat from the production of biomass precursors towards energy metabolism, as the fraction of NADH produced via the TCA cycle increases from 8.1% to 12.9%. This is also reflected in the lowered fractions of 2-oxoglutarate (31% vs. 50%) and oxaloacetate (35% vs. 46%) branching from the TCA cycle towards anabolic pathways.

Metabolite flux minimisation revealed a potential problem in the model by predicting that flux through extracellular protons is not essential for optimal growth: Excess intracellular protons, which are transported to the periplasm via the complexes of the electron transfer chain, can be converted to water by ferroxidase in the periplasm instead of being exported through the outer membrane. The electrons required for the involved reduction of molecular oxygen are donated by Fe^2+^, which has to be imported at an extreme rate of 14 mmol gDW^−1^ h^−1^, which is equivalent to 0.8 grams of iron per gram dry weight per hour. This is obviously unrealistic, especially as the produced Fe^3+^ has a low solubility at neutral pH [55]. The only reason why the free import of electrons resulting from the free import of Fe^2+^ does not have more pronounced effects on the model as a whole is that it is limited to the periplasm, i.e. these electrons cannot be used to perform work in the cytosol. To limit the flux through the ferroxidase reaction, the secretion rate of Fe^3+^ should be restricted to zero (or a value close to zero).

We demonstrated the ability of split-ratio analysis and branch point analysis to assign metabolic roles. These roles are often in agreement with long-established knowledge of metabolic pathways, but in other cases it becomes obvious that the metabolic roles are strongly modified in different contexts and that the metabolism is highly flexible in making use of different capabilities many metabolites provide.

In the small number of discussed examples, the role of L-glutamine, L-glutamate, and L-aspartate as amino-group donors became obvious. Furthermore, the forced acetate secretion flux was shown to be responsible for more than 90% of the total acetate-producing flux. Thioredoxin was predicted to be redundant in steady-state metabolism despite being involved in ten reactions in the network. This prediction is in agreement with the observation that *trxA trxC* double mutants of *E. coli* are phenotypically inconspicuous [56]. However, thioredoxins may be essential for the oxidative stress response [57], which is an inherently non-steady-state process and thus cannot be studied using FBA.

It should be noted that the transformation of reaction fluxes to metabolite fluxes and split ratios and the computational methods introduced here are applicable to any flux distribution, whether computationally predicted or experimentally determined, e.g. via ^13^C tracer analysis. The only requirement is a flux distribution at the resolution of individual reactions rather than summed “pathway fluxes”.

## Conclusions

This paper introduces a new, metabolite-centric view on flux distributions in genome-scale metabolic networks. We have shown how a metabolite-centric view on flux distributions opens the door to a more detailed analysis than is possible within the traditional reaction-centric framework. Split-ratio analysis and its extension branch point analysis facilitate the interpretation of flux distributions by providing metabolic context.

Within a metabolite-centric view, it is very easy to simultaneously analyse all fluxes in a metabolic context, as demonstrated here for the full metabolic contexts of energy metabolism, the TCA cycle, the interconnected transamination cycles, the superpathway of glycolysis and the pentose phosphate pathway, and the folate-dependent one-carbon pool. This contextualisation leads to new biological interpretations not apparent from isolated reaction fluxes, which can be used for validating and refining the model’s predictive capabilities for specific growth scenarios.

In summary, switching from a reaction-centric to a metabolite-centric view on flux distributions allows a wealth of inferences to be drawn that are not apparent from the reaction fluxes alone. The computational methods we have introduced here allow the analysis of reaction fluxes, whether predicted or determined experimentally, in context rather than in isolation.

## Methods

### Metabolic model iJO1366

The genome-scale metabolic model iJO1366 covers 1366 open reading frames of *Escherichia coli* K-12 MG1655. The reconstructed network comprises a total of 2583 reaction nodes (including transport and exchange reactions and two artificial reactions modelling the formation of biomass) and 1805 metabolite nodes (1039 cytoplasmic, 442 periplasmic, 324 extracellular). The model was downloaded from the supplementary material of the corresponding publication [10] in the SBML format [58].

iJO1366 is a stoichiometric model, which is mathematically represented by a stoichiometric matrix **S** and a set of lower and upper flux bounds **lb** ≤ **v** ≤ **ub** constraining the flux variables **v**[59]. **S** is an *m* × *n* matrix, where *m* is the number of metabolites (1805), *n* is the number of reactions in the network (2583), and **v** is an *n* × 1 vector. The entry *s*_*ij*_ of **S** is the stoichiometric coefficient of metabolite *i* in reaction *j*. It is positive if metabolite *i* occurs on the right-hand side of reaction *j*, negative if it occurs on the left-hand side, and zero if it is not involved in the reaction. The flux bounds {**lb**, **ub**} represent, inter alia, thermodynamic constraints (e.g. irreversibility of reactions) and medium properties (e.g. constraining the uptake of a compound not present in the medium to zero).

Important model parameters are the *growth-associated* and *non-growth-associated maintenance requirements* (abbreviated as GAM and NGAM, respectively). The GAM is part of the biomass reaction and models the ATP cost of growth-associated processes, most importantly DNA, RNA, and protein polymerisation, per gram dry weight, while the NGAM is modelled as an ATP hydrolysis reaction with a fixed flux and represents maintenance processes that consume ATP but are not associated with growth [60]. These processes include, among others, DNA repair and maintaining turgor pressure and membrane potential.

### Flux balance analysis (FBA)

The fluxes through the network in steady state were computed using flux balance analysis [16,61,62]. FBA is a mathematical method for predicting the flux distribution in a stoichiometric network without requiring knowledge of *in-vivo* kinetic parameters. Its underlying assumptions are steady state and optimality. The first assumption, mathematically expressed as **Sv** = 0, is that the system is in a flux equilibrium with all metabolite concentrations constant [63]. The second assumption is that the organism has been optimised through its evolutionary history for some biological goal [17], usually biomass production (growth).

We used the implementation of FBA in *metano*, the open-source software toolkit developed in our group (Riemer et al., manuscript in preparation). The software is written in the Python programming language and is available online (http://metano.tu-bs.de). We verified our implementation by comparing our solutions for the earlier model iAF1260 to the corresponding published FBA solutions [11], using identical simulation parameters in two different scenarios (growth on glucose minimal medium; scenario 1: glucose uptake rate 8 mmol gDW^−1^ h^−1^, carbon-limited; scenario 2: glucose uptake rate 11 mmol gDW^−1^ h^−1^, oxygen uptake rate 18.2 mmol gDW^−1^ h^−1^, carbon- and oxygen-limited). Apart from alternate (but also optimal) pathway usage, both FBA solutions could be reproduced (data not shown).

### Simulation parameters

For the analyses on the model iJO1366, the glucose uptake rate was set to 11.0 mmol gDW^−1^ h^−1^, and acetate secretion was added at a rate of 6.4 mmol gDW^−1^ h^−1^. These values were reported in [49] for exponential growth in a bioreactor, the scenario that corresponds best to the infinite environment assumed implicitly in FBA. Moreover, the specific constraints described in [11] for aerobic growth on glucose were employed. These include blocking reactions that are not active in this scenario and fixing the effective proton translocation rate for the NADH dehydrogenase complex at 1.5 protons per molecule of NADH. The latter is equivalent to setting the ratio between proton-translocating and non-proton-translocating NADH dehydrogenase to 1:1. The *metano* input files for this scenario and the resulting output files are provided in Additional files 2 and 3, respectively.

A second scenario, which was derived from the one described above by adding a forced flux of 5 mmol gDW^−1^ h^−1^ through proton-translocating transhydrogenase, was employed in an attempt to resolve discrepancies found initially between model predictions and experimental data.

### Flux variability analysis (FVA)

The solutions returned by FBA are not necessarily unique, as for whole-genome models, the objective function usually assumes the optimum at more than one point in the solution space [60]. Flux variability analysis successively minimises and maximises each flux variable under the additional constraint of an optimal or suboptimal objective function value [64], thus exploring the shape of the optimal solution space. In the present study, FVA was used to test whether reported fluxes or split ratios must necessarily assume the values predicted by FBA or if there are alternate optimal solutions. FVA has been implemented in *metano* in the ‘fast FVA’ variety of the algorithm [53].

### Split-ratio analysis

We developed the following algorithm to compute metabolite fluxes and split ratios from reaction fluxes:

Given a stoichiometric matrix **S** and a flux vector **v**,

For each metabolite *i:Φ*_*i*_ is the total flux through metabolite *i* (or flux sum, as defined in [32]), and the $\rho_{\mathrm{ij}}^{*}$ are the split ratios of metabolite *i*, which are again grouped by sign (producing or consuming) and expressed as percentages of *Φ*_*i*_. More precisely, $\rho_{\mathrm{ij}}^{*}$ is the fraction of metabolite *i* produced (if positive) or consumed (if negative) via reaction *j*.

1. Compute partial fluxes *ρ*_*ij*_ = *s*_*ij*_ · *v*_*j*_ for all reactions *j* and group into positive (producing) and negative (consuming):

$$P_{i}=\left\{j\left|\rho_{\mathrm{ij}}>0\right)\right\},C_{i}=\left\{j\left|\rho_{\mathrm{ij}}<0\right)\right\}$$

2. Compute:

$$\Phi_{i}=\sum_{j\in P_{i}}\rho_{\mathrm{ij}}=-\sum_{j\in C_{i}}\rho_{\mathrm{ij}}$$

and divide partial fluxes by this flux sum:

$$\rho_{i}^{*}=\frac{\rho_{\mathrm{ij}}}{\Phi_{i}}$$

### Branch point analysis

Branch point analysis was developed to generalise the approach of split-ratio analysis to the whole metabolic network and visualise the extended metabolic context of a selected metabolite or reaction. The analysis starts with the complete bipartite reaction graph, which consists of all reactions and metabolites that are active in the given FBA solution (i.e. carry a flux above some threshold). As the complete graph is much too complex for a meaningful interpretation, the graph is reduced according to user-supplied parameters.

The user selects a node (i.e. reaction or metabolite) as the hub of the analysis. Optionally, the user can provide a list of nodes to be excluded and a list of nodes to be disconnected. Excluded nodes are completely removed from the graph, while disconnected nodes are split into separate instances for each occurrence of the metabolite or reaction in the network. Typically, the first list would be used to exclude metabolites that are ubiquitous, but in most contexts uninteresting to the user (e.g. H_2_O or H^+^). The second list can be used to prevent crowding of the graph by promiscuous compounds such as currency metabolites (e.g. ATP or NADH). Finally, the user can specify a maximum distance to the selected hub node. Nodes beyond this limit will not be shown in the final graph.

It is possible to select more than one hub for the analysis, which allows fine-grained selection of nodes for inclusion and exclusion. However, if the resulting graph is disconnected, only the largest connected component is displayed.

**Figure 6:**
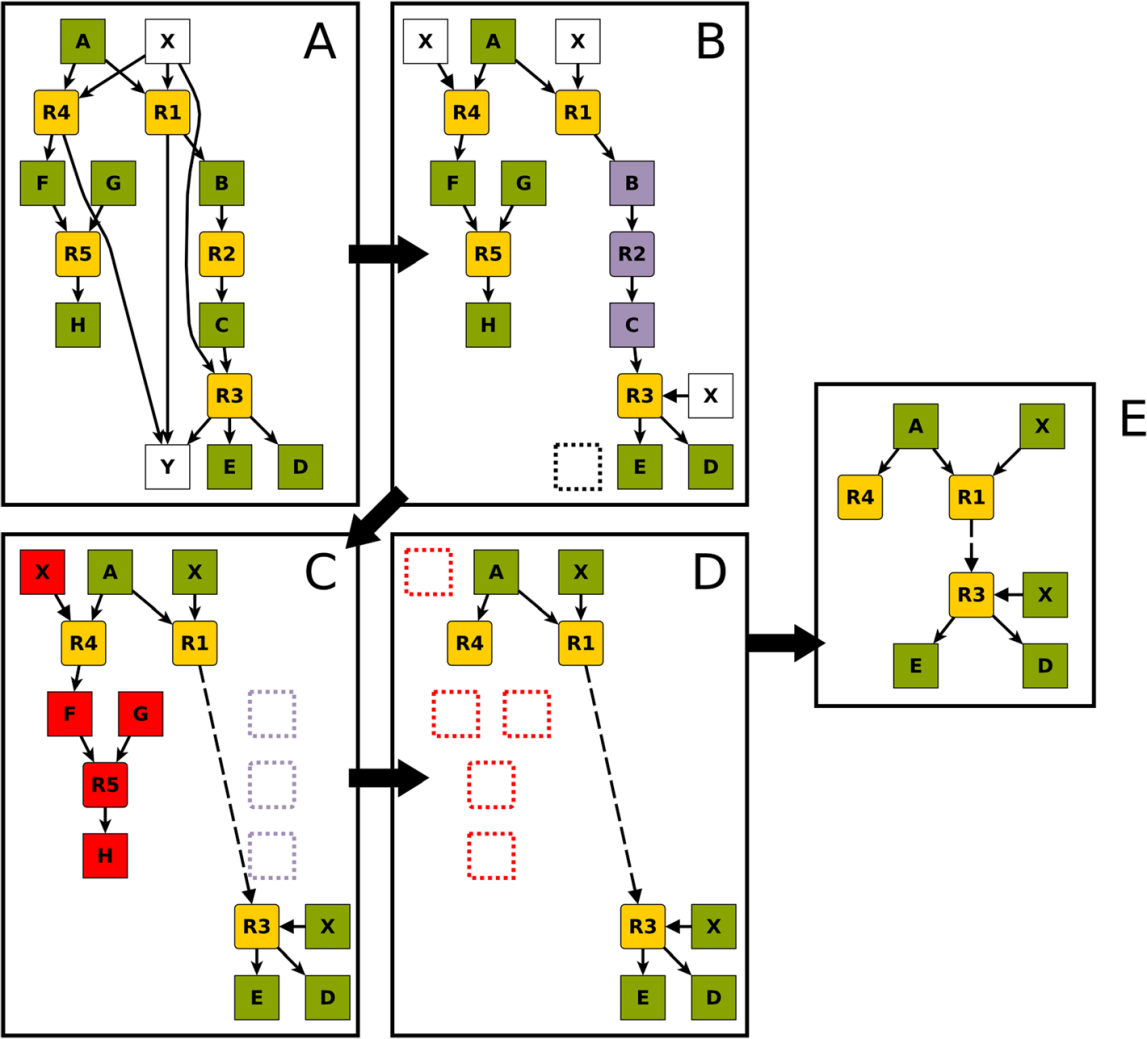
**Sequence of example graphs demonstrating the algorithm of branch point analysis.** Node R1 has been selected as hub, node X is to be disconnected, and Y is to be removed. The configured maximum distance from the selected node is two. **A**) Initial bipartite graph. **B**) Disconnection (X) or removal (Y), respectively, of selected nodes. **C**) Bridging of transitory nodes (B, R2, C). **D**) Removal of all nodes with a distance greater than two from the hub, R1. **E**) Condensed metabolic context of reaction R1 produced by branch point analysis.

The algorithm is illustrated schematically in Figure 6. Branch point analysis starts with the calculation of the split ratios for all metabolites based on the given flux distribution. Subsequently, the bipartite reaction graph is built, and the edges are labelled with their corresponding split ratios (as percentage of the flux through the adjacent metabolite node). Figure 6A shows the initial state of an example graph representing the metabolic context of reaction R1, with edge labels omitted for clarity. Next, the respective nodes specified by the user are removed and disconnected (Figure 6B). At this point, the complexity of the graph is further reduced by bridging transitory nodes (Figure 6C). Specifically, each node that has exactly two neighbours is replaced by a dashed edge. This step is applied iteratively until no such nodes remain. Disconnected nodes are not taken into account when the number of neighbours is determined. Finally, all nodes beyond the specified maximum distance are removed (Figure 6D). Besides the selected node, the remaining graph contains only nodes at which the metabolic flux branches – metabolic branch points (Figure 6E).

We implemented branch point analysis in the Python programming language based on *metano*. The resulting program, AMEBA (Advanced MEtabolic Branchpoint Analysis), is distributed under the GNU General Public License version 3 and can be downloaded from http://metano.tu-bs.de/ameba. Our implementation uses NetworkX [65] for graph operations and Graphviz [66] for graph layouting. An interactive mode has been implemented using the xdot module (http://code.google.com/p/jrfonseca/wiki/XDot).

### Metabolite flux minimisation

The following algorithm was used to determine the minimum flux that each metabolite node has to carry under the constraint of (sub)optimal biomass production:

1. Split flux variables into positive and negative components:

$$v_{j}=v_{j}^{+}-v_{j}^{-},\;\mathrm{where}\;v_{j}^{+}\geq 0\;\mathrm{and}\;v_{j}^{-}\geq 0,$$

and duplicate and negate the corresponding columns of the stoichiometric matrix **S**. The result is a vector of non-negative flux variables. Lower and upper bounds have to be adjusted accordingly. In practice, flux variables constrained to zero can be removed along with the corresponding columns of **S**.

2. Using the modified flux vector and stoichiometric matrix from step 1:

For each metabolite *i*, solve the following convex linear optimisation problem:

$$\text{Minimise}\sum_{j}s_{\mathrm{ij}}^{+}\cdot v_{j},\mathrm{where}\;s_{\mathrm{ij}}^{+}=\left\{\begin{array}{c} s_{\mathrm{ij}},s_{\mathrm{ij}}\geq 0\\ 0,\mathrm{else} \end{array}\right)$$

in steady state: **Sv** = 0

with inequality constraints **lb** ≤ **v** ≤ **ub**

and the additional constraint:

$$v_{\text{Biomass}}\geq \zeta \cdot v_{\text{Biomass,opt}}\;\text{with}\;0<\zeta \leq 1\text{.}$$

In effect, this algorithm successively minimises each of the producing metabolite flux sums *Φ*_*i*_. The optimisation problems were solved using the GNU Linear Programming Kit (GLPK; http://www.gnu.org/software/glpk/glpk.html). As metabolite flux minimisation is very similar to FVA, the ‘fast FVA’ strategy is applicable to this algorithm as well, and it has been implemented in *metano* with this performance optimisation. In this study, metabolite flux minimisation was used for identifying nonessential metabolites and for determining minimal metabolite turnover rates *Φ*_*i.*_

## Abbreviations

CoA: Coenzyme A; DHA: Dihydroxyacetone; F6P: Fructose 6-phosphate; FBA: Flux balance analysis; FVA: Flux variability analysis; G6P: Glucose 6-phosphate; GAM: Growth-associated maintenance; ICD: Isocitrate dehydrogenase; NGAM: Non-growth-associated maintenance; PEP: Phosphoenolpyruvate; TCA cycle: Tricarboxylic acid cycle; THFA: Tetrahydrofolate.

## Competing interests

The authors declare that they have no competing interests.

## Authors’ contributions

SAR and RR developed and implemented the new methods described here (SAR: split-ratio analysis and metabolite flux minimisation, RR: branch point analysis), carried out the *in-silico* study on the *E. coli* model, and drafted the manuscript. SAR and DS conceived of the study. DS participated in the design and coordination of the study and helped to draft the manuscript. All authors read and approved the final manuscript.

## Supplementary Material

Additional file 1: Figure S1 Metabolite fluxes and split ratios within the superpathway of glycolysis and the pentose phosphate pathway.Green: Metabolite nodes, yellow: enzyme nodes. Numbers in metabolite nodes are total flux in mmol gDW^−1^ h^−1^. Edge labels are split ratios as fractions of the flux through the adjacent metabolite node. Click here for file

Additional file 2 **Metano input files generated from model iJO1366.** Model and scenario file – see http://metano.tu-bs.de/quickref.html for description of file formats. Click here for file

Additional file 3 **Metano output files (FBA, FVA, split-ratio analysis).** See http://metano.tu-bs.de/quickref.html for description of file formats. Click here for file
