# Supplementary figures and images for: A metabolite-centric view on flux distributions in genome-scale metabolic models
Source: BMC Syst Biol. 2013 Apr 12;7:33. doi: 10.1186/1752-0509-7-33 (PMC3644240; doi:10.1186/1752-0509-7-33)

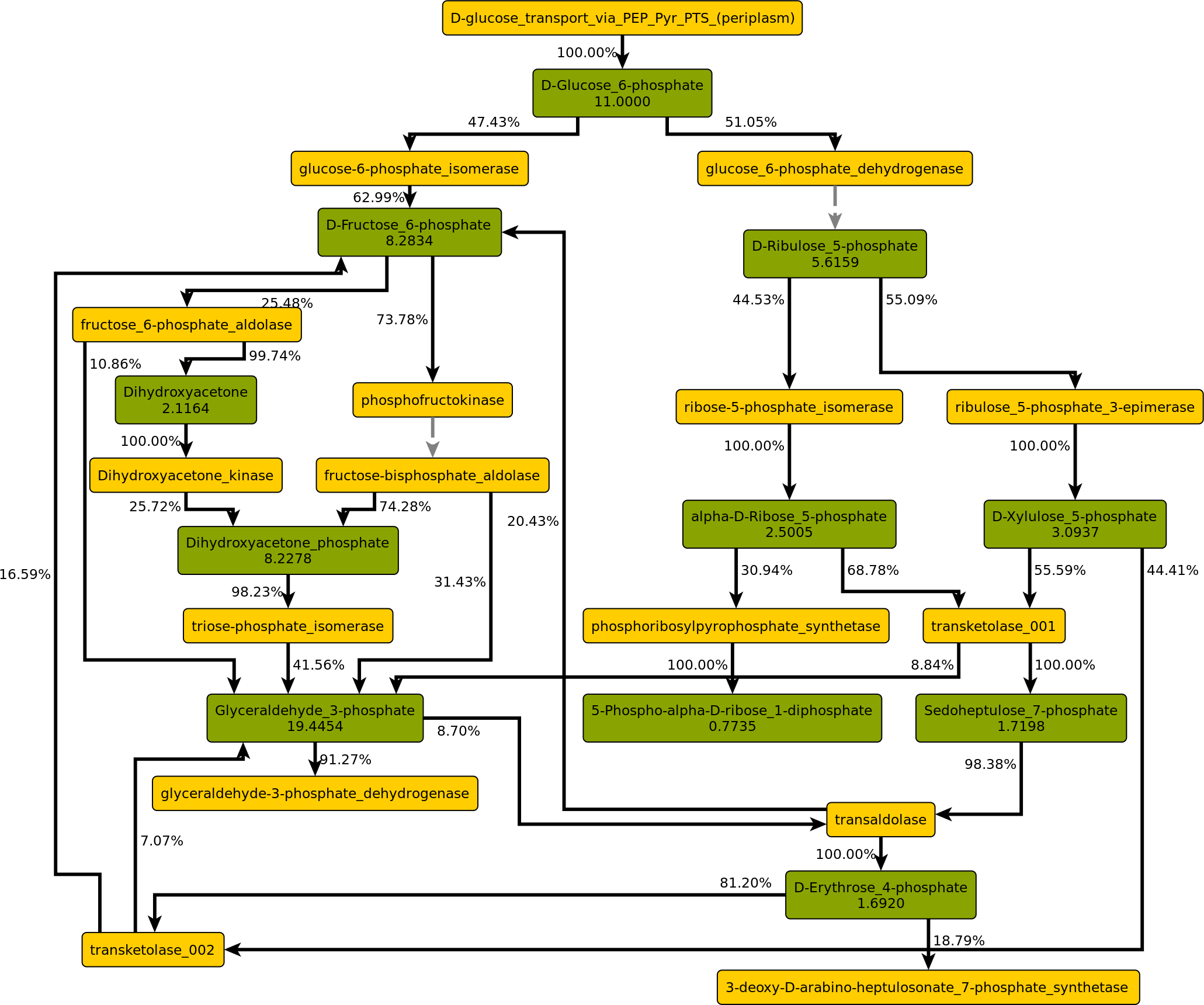

Supplement: Additional file 1: Figure S1 — Metabolite fluxes and split ratios within the superpathway of glycolysis and the pentose phosphate pathway.Green: Metabolite nodes, yellow: enzyme nodes. Numbers in metabolite nodes are total flux in mmol gDW−1 h−1. Edge labels are split ratios as fractions of the flux through the adjacent metabolite node. [file 1752-0509-7-33-S1.tiff]
